# The population attributable fraction of low education for mortality in South Korea with improvement in educational attainment and no improvement in mortality inequalities

**DOI:** 10.1186/s12889-015-1665-x

**Published:** 2015-03-31

**Authors:** Dohee Lim, Kyoung Ae Kong, Hye Ah Lee, Won Kyung Lee, Su Hyun Park, Sun Jung Baik, Hyesook Park, Kyunghee Jung-Choi

**Affiliations:** Department of Preventive Medicine, Ewha Womans University School of Medicine, 911-1, Mok-6dong, Yang Cheon-gu, Seoul 158-710 South Korea; Clinical Trial Center, Ewha Womans University Medical Center, 911-1, Mok-6dong, Yang Cheon-gu, Seoul 158-710 South Korea; Department of Social and Preventive Medicine, Inha University School of Medicine, Incheon, South Korea

**Keywords:** Educational attainment, Mortality inequality, Population Attributable Fraction

## Abstract

**Background:**

The educational attainment of Koreans has greatly increased, which was expected to reduce the magnitude of the population attributable fraction (PAF) of mortality associated with low education levels. However, increase in the relative risk (RR) of mortality among those with lower educational levels actually increased the PAF. The purpose of this study was to examine the change in the PAF of lower educational levels for mortality in Korea, where educational attainment has improved and is associated with the exacerbation of inequalities in mortality levels.

**Methods:**

National census data were used to derive educational levels. The mortality-associated RR of lower educational levels was calculated by reference to national census and death certificate data from 1995, 2000, 2005, and 2010. PAFs were calculated for all-cause mortality, malignant neoplasms, cerebrovascular disease, heart disease, and suicide by gender and age group (30–44 and 45–59 years).

**Results:**

The PAF of low educational level in terms of total mortality has decreased since 1995 in both genders. This trend was more prominent among those aged 30–44 years. However, the PAFs of suicide in younger females (30–44 years) and of cerebrovascular disease in older males (45–59 years) have increased. The RRs of all-cause mortality and those of the four leading causes of death in those with the lowest educational levels have increased, especially in females aged 30–44 years.

**Conclusions:**

The consistent and sharp increase in the attainment of education has contributed to the reduction in the PAFs of lower education for mortality, despite the fact that mortality inequalities have not improved. Efforts to reduce health inequalities must promote healthy public policy and address public health policies.

**Electronic supplementary material:**

The online version of this article (doi:10.1186/s12889-015-1665-x) contains supplementary material, which is available to authorized users.

## Background

Many empirical studies have assessed the influence of socioeconomic position (SEP) on health using various measures of health inequality [1-3] such as the relative risk (RR), risk difference (RD), the slope index of inequality (SII), and the relative index of inequality (RII). While measures such as these are often applied to assess the magnitude of excess risks, the population attributable fraction (PAF) is used to quantify the attributable burden of exposure on a population, as it considers both the magnitude of excess risks along with the prevalence of a given exposure within the population [4]. In terms of SEP, the PAF is interpreted as the potential improvement in the health of a population if all subgroups were to achieve the ideal scenario for that particular factor. Such a measure is preferable to other common metrics, such as the RII, as it is a more intuitive measure of improvement, while still accounting for changes in subgroup size [5]. Measuring the PAF of lower socioeconomic groups can therefore be used to make projections in regard to potential population interventions seeking to reduce health inequalities among groups [6,7]; however, few studies have investigated the PAFs of health outcomes in relation to SEP.

Education is one of the most commonly used indicators of SEP on the basis of Weberian theory, as it been shown to affect not only future occupational opportunities and earning potential, but also the ability to understand health information [8]. Policies that increase an individual’s educational attainment would therefore be expected to improve health outcomes [7,9]. Over the past 60 years, the Korean government has implemented a wide range of reforms aimed at extending educational opportunities to all segments of society, resulting in substantial improvements in educational attainment [10]. Such improvements should be expected to have resulted in a reduced PAF for mortality among lower education groups.

In addition to educational improvements, Korea has experienced a rapid increase in life expectancy, which stands at 78.5 years in 2005, a level similar to that of other developed countries [11]. However, many studies have reported socioeconomic inequalities in mortality, both in Korea as well as other developed countries [12-16]. Indeed, these inequalities have increased according to recent studies [17,18]. The increase in the RR of mortality among those with less education is expected to contribute to an increase in the magnitude of the PAF of lower education levels.

Therefore, the purpose of this study was to examine the change in the PAF of lower education for mortality in Korea, where educational attainment has steadily improved and the inequalities in mortality rates have been somewhat exacerbated.

## Methods

### Data and subjects

Calculation of the PAF requires prevalence data for all levels of educational attainment, along with the RRs of lower educational levels. Information regarding the prevalence of educational attainment was based on national census data obtained from Statistics Korea reports for 1995, 2000, 2005, and 2010 [19]. National census data are accessible electronically through the Korean Statistical Information Service (KOSIS). The overall response rates for questions regarding education were excellent, with only 0.03% of men and 0.02% of women failing to provide responses in 1995 and 2000; these levels dropped to near 0% in both 2005 and 2010. Death certificate data and national census data were used for calculating the RRs of lower educational level groups. Death statistics are based on death certificate forms, which are filled out in accordance with the Family Registration Law and Statistics Law. Death certificates are filed following the death of any individual over the age of 1, with records dating back to the mid-1980s [20]. The reliability of educational level details within the death certificate data has been reported to be substantial [21].

Inclusion in this study was limited to adults aged 30–59 years. Subjects <30 years of age were excluded so as to allow sufficient time for the completion of a college education, while subjects >60 years of age were excluded due to inherent differences in SEP associated with each educational category relative to younger adult groups. The subjects were divided into two age groups: younger adults (aged 30–44 years) and older adults (aged 45–59 years). The total study population included 47,969,747 younger adults (50.6% men and 49.4% women), 74,272 of whom died, and 33,385,087 older adults (49.9% men and 50.1% women), 273,936 (72.5% men and 27.5% women) of whom died. This study was approved by the Ewha Medical Center Institutional Review Board, Seoul, Korea (ECT 14-25A-37 [2014.08.06]).

### Educational attainment

Educational attainment was used as the primary indicator of SES. The levels of educational attainment were categorized as middle school graduate or less (ISCED 1 and 2), high school graduate (ISCED 3), and college graduate or higher (ISCED 5); ISCED 4 was not included in this study because no equivalent level exists in Korea [22]. ISCED 0 was not considered in this study, as it is not a requisite for entry into subsequent levels of schooling in Korea.

### Causes of death

The causes of death listed on death certificates were coded according to the 6th Korean Standard Classification of Diseases (KCD-6), as codified based on the 10th International Classification of Diseases (ICD-10). Data were analyzed for all-cause mortality, along with the four leading causes of death in 2012 (from highest to lowest): malignant neoplasms (C00-C97), cerebrovascular disease (I60-I69), heart disease (I20-I51), and suicide (X60-X84).

### Statistical analysis

Mortality rates were calculated using the population of a given age group in a given census year (1995, 2000, 2005, and 2010) relative to the total number of moralities within that age group over the same period. Each number was driven from national census data and death certificate data, respectively. Mortality rates were calculated based on direct standardization methods using the World Health Organization (WHO) world population as the standard population. Age-adjusted mortality rates by gender for the main causes of death were also calculated.

PAF were calculated using the PIF (potential impact fraction) equation [23], with those who had at least graduated from college treated as the counterfactual level. The RR for mortality was calculated for high school graduates and those with middle school graduate or less, and separated by gender and age group. Log-linear regression was used to estimate the RRs and 95% confidence intervals (CIs) for mortality. This measurement can be used for ordered or non-ordered groups and can take into account subgroups of different sizes [24].

$$ PIF=\frac{{\displaystyle \sum_{i=1}^n{P}_iR{R}_i}-{\displaystyle \sum_{i=1}^n{P}_i^{\prime }R{R}_i}}{{\displaystyle \sum_{i=1}^n{P}_iR{R}_i}} $$

RR_i_: relative risk at exposure level i

P^'^_i_: counterfactual distribution of exposure

P_i_: population distribution of exposure

n: maximum exposure level

## Results

Table 1 shows the increase in educational attainment from 1995 to 2010. In 1995, a plurality of younger adults completed high school (47.3% men, 48.1% women); however, the majority of individuals were college graduates in 2010 (56.2% men, 48.8% women). In contrast, the proportion of the population with the lowest educational attainment, middle school graduate or less, decreased substantially. In 2010, only 4% of younger adults had, at most, graduated from middle school, whereas this figure was 27% in 1995. The educational attainment of older adults also improved, but not nearly as much as that of younger adults. Until 2000, it was most common for older adults to have finished middle school at most.

**Table 1:** **Educational attainment prevalence for men and women by age groups, South Korea 1995-2010**

**Thousand people (%)**																
	**1995**				**2000**				**2005**				**2010**			
	**Total**	**Middle-school graduate or less**	**High-school graduate**	**College graduate or higher**	**Total**	**Middle-school graduate or less**	**High-school graduate**	**College graduate or higher**	**Total**	**Middle-school graduate or less**	**High-school graduate**	**College graduate or higher**	**Total**	**Middle-school graduate or less**	**High-school graduate**	**College graduate or higher**
Men																
30-44	5,829	1,167 (20.0)	2,760 (47.3)	1,900 (32.6)	6,215	822 (13.2)	2,939 (47.3)	2,451 (39.4)	6,208	393 (6.3)	2,727 (43.9)	3,088 (49.7)	5,998	235 (3.9)	2,395 (39.9)	3,368 (56.2)
45-59	3,214	1,532 (47.7)	1,105 (34.4)	576 (17.9)	3,641	1,493 (41.0)	1,405 (38.6)	741 (20.4)	4,515	1,355 (30.0)	1,926 (42.7)	1,234 (27.3)	5,293	1,221 (23.1)	2,320 (43.8)	1,753 (33.1)
30-59	9,043	2,699 (29.9)	3,865 (42.7)	2,476 (27.4)	9,856	2,315 (23.5)	4,344 (44.1)	3,192 (32.4)	10,723	1,748 (16.3)	4,653 (43.4)	4,322 (40.3)	11,291	1,455 (12.9)	4,715 (41.8)	5,121 (45.4)
Women																
30-44	5,606	1,977 (35.3)	2,696 (48.1)	932 (16.6)	6,061	1,323 (21.8)	3,291 (54.3)	1,446 (23.9)	6,124	551 (9.0)	3,293 (53.8)	2,280 (37.2)	5,928	236 (4.0)	2,798 (47.2)	2,894 (48.8)
45-59	3,228	2,458 (76.2)	597 (18.5)	171 (5.3)	3,630	2,400 (66.1)	954 (26.3)	275 (7.6)	4,519	2,255 (49.9)	1,711 (37.9)	553 (12.2)	5,345	1,972 (36.9)	2,434 (45.5)	939 (17.6)
30-59	8,833	4,435 (50.2)	3,293 (37.3)	1,103 (12.5)	9,691	3,723 (38.4)	4,245 (43.8)	1,721 (17.8)	10,643	2,806 (26.4)	5,005 (47.0)	2,832 (26.6)	11,273	2,207 (19.6)	5,232 (46.4)	3,833 (34.0)

Age-adjusted mortality rates, along with the magnitude of these inequalities relative to educational level are shown in Tables 2 and 3. The most pronounced inequalities were found among men aged 30–44 years (Table 2), including the RRs for total mortality of >8.0 for middle school graduates or less from 1995 to 2010. The RRs of middle-school graduates or less for cerebrovascular disease, heart disease, and suicide were also higher at >6.0 in 2010, although the RRs for cerebrovascular disease and suicide did decrease between 1995 and 2010. In contrast, the mortality rates for suicide showed a modest increase across all educational levels over the period of this study.

**Table 2:** **Age-adjusted mortality and relative risks of all-cause and four causes of death according to educational level in men**

	**All-cause**		**Malignant neoplasms**		**Cerebrovascular**		**Heart disease**		**Suicide**	
	**Mortality (95% CI)**	**RR (95% CI)**	**Mortality (95% CI)**	**RR (95% CI)**	**Mortality (95% CI)**	**RR (95% CI)**	**Mortality (95% CI)**	**RR (95% CI)**	**Mortality (95% CI)**	**RR (95% CI)**
30 ~ 44 years old										
1995										
Middle-school graduate or less	832.8 (815.4, 850.1)	8.0 (7.6, 8.4)	96.5 (90.9, 102.1)	4.2 (3.7, 4.7)	39.8 (36.1, 43.5)	7.7 (6.1, 9.7)	57.1 (52.6, 61.7)	6.9 (5.8, 8.3)	54.3 (49.7, 59.0)	9.0 (7.4, 11.0)
High-school graduate	201.4 (196.0, 206.8)	2.0 (1.9, 2.1)	34.7 (32.4, 36.9)	1.5 (1.4, 1.7)	9.8 (8.6, 11.1)	2.0 (1.6, 2.6)	12.6 (11.3, 14.0)	1.6 (1.3, 1.9)	12.6 (11.3, 14.0)	2.2 (1.8, 2.7)
College graduate or higher	103.5 (98.7, 108.4)	1.0	23.0 (20.6, 25.3)	1.0	4.7 (3.6, 5.8)	1.0	8.0 (6.6, 9.4)	1.0	5.6 (4.5, 6.7)	1.0
2000										
Middle-school graduate or less	810.3 (787.9, 832.7)	8.1 (7.7, 8.5)	96.0 (88.8, 103.2)	3.6 (3.2,4.0)	42.2 (37.2, 47.1)	6.9 (5.6, 8.4)	47.3 (42.1, 52.5)	6.7 (5.6, 8.0)	89.5 (81.5, 97.4)	9.1 (7.8, 10.7)
High-school graduate	195.3 (190.3, 200.4)	2.0 (2.0, 2.2)	34.2 (32.1, 36.3)	1.4 (1.2,1.5)	10.3 (9.2, 11.5)	1.8 (1.5, 2.2)	13.6 (12.3, 14.9)	2.0 (1.6, 2.3)	21.1 (19.4, 22.7)	2.3 (2.0, 2.7)
College graduate or higher	96.0 (92.1, 100.0)	1.0	25.3 (23.3, 27.3)	1.0	5.6 (4.6, 6.6)	1.0	6.6 (5.5, 7.6)	1.0	8.9 (7.7, 10.1)	1.0
2005										
Middle-school graduate or less	814.1 (782.6, 845.5)	9.3 (8.8, 9.8)	87.6 (78.2, 97.0)	4.2 (3.7, 4.7)	37.5 (31.1, 43.8)	6.9 (5.6, 8.6)	43.6 (36.2, 50.9)	5.0 (4.1, 6.1)	146.6 (132.6, 160.6)	9.3 (8.2, 10.5)
High-school graduate	189.7 (184.6, 194.8)	2.3 (2.1, 2.4)	31.4 (29.3, 33.5)	1.5 (1.4, 1.7)	10.8 (9.6, 12.0)	2.2 (1.8, 2.6)	13.1 (11.8, 14.5)	1.6 (1.4, 1.9)	35.6 (33.3, 37.9)	2.3 (2.1, 2.6)
College graduate or higher	84.3 (81.1, 87.6)	1.0	21.1 (19.4, 22.7)	1.0	5.2 (4.3, 6.0)	1.0	8.0 (7.0, 9.0)	1.0	15.5 (14.1, 16.9)	1.0
2010										
Middle-school graduate or less	708.7 (673.1, 744.2)	8.4 (8.0, 8.9)	72.6 (61.5, 83.7)	3.8 (3.3, 4.5)	23.0 (16.9, 29.0)	6.0 (4.5, 8.0)	45.4 (36.4, 54.4)	7.1 (5.7, 8.9)	163.5 (145.1, 181.9)	6.9 (6.1, 7.8)
High-school graduate	186.9 (181.4, 192.3)	2.2 (2.1, 2.3)	28.1 (26.1, 30.2)	1.6 (1.4, 1.7)	8.4 (7.3, 9.6)	2.1 (1.7, 2.6)	11.2 (9.9, 12.6)	1.8 (1.5, 2.2)	52.5 (49.5, 55.5)	2.2 (2.0, 2.4)
College graduate or higher	84.3 (81.2, 87.4)	1.0	18.3 (16.8, 19.7)	1.0	3.8 (3.1, 4.4)	1.0	6.1 (5.2, 6.9)	1.0	23.0 (21.3, 24.6)	1,0
45 ~ 59 years old										
1995										
Middle-school graduate or less	1551.8 (1534.6, 1569.1)	2.4 (2.3, 2.5)	477.6 (468.2, 487.1)	1.8 (1.7, 1.9)	165.7 (160.1, 171.2)	1.9 (1.7, 2.1)	98.6 (94.2, 102.9)	1.7 (1.5, 1.9)	35.1 (32.4, 37.7)	3.1 (2.5, 4.0)
High-school graduate	899.4 (881.0, 917.8)	1.4 (1.3, 1.4)	318.6 (307.5, 329.7)	1.2 (1.1, 1.3)	120.4 (113.5, 127.3)	1.3 (1.2, 1.5)	71.1 (66.0, 76.3)	1.2 (1.0, 1.3)	18.3 (15.8, 20.8)	1.6 (1.2, 2.0)
College graduate or higher	661.1 (640.2, 682.0)	1.0	264.2 (251.0, 277.5)	1.0	88.9 (81.2, 96.7)	1.0	61.0 (54.6, 67.4)	1.0	11.2 (8.5, 13.8)	1.0
2000										
Middle-school graduate or less	1381.6 (1365.4, 1397.8)	2.4 (2.3, 2.5)	434.5 (425.5, 443.4)	1.8 (1.7, 1.8)	141.8 (136.7, 146.9)	2.2 (2.0, 2.4)	91.0 (86.8, 95.1)	1.5 (1.4, 1.7)	51.8 (48.6, 55.1)	3.8 (3.1, 4.6)
High-school graduate	804.4 (789.4, 819.3)	1.4 (1.3, 1.4)	294.2 (285.1, 303.3)	1.2 (1.1, 1.3)	92.0 (86.8, 97.1)	1.4 (1.3, 1.5)	72.2 (67.7, 76.6)	1.2 (1.1, 1.3)	25.9 (23.3, 28.5)	1.9 (1.5, 2.3)
College graduate or higher	600.1 (582.3, 617.9)	1.0	250.0 (238.5, 261.5)	1.0	67.6 (61.5, 73.7)	1.0	62.5 (56.7, 68.3)	1.0	14.2 (11.5, 6.9)	1.0
2005										
Middle-school graduate or less	1248.5 (1232.2, 1264.7)	3.1 (3.0, 3.2)	396.9 (387.9, 405.8)	2.1 (2.0, 2.2)	100.1 (95.6, 104.6)	2.7 (2.4, 3.0)	79.3 (75.2, 83.4)	1.9 (1.7, 2.1)	97.6 (92.9, 102.3)	4.1 (3.6, 4.6)
High-school graduate	642.8 (631.2, 654.4)	1.6 (1.6, 1.7)	247.5 (240.1, 254.8)	1.3 (1.3, 1.4)	57.4 (53.9, 61.0)	1.5 (1.4, 1.7)	53.9 (50.5, 57.2)	1.3 (1.2, 1.5)	42.7 (39.8, 45.6)	1.8 (1.6, 2.0)
College graduate or higher	427.9 (415.0, 440.7)	1.0	190.6 (182.0, 199.2)	1.0	39.1 (35.2, 3.1)	1.0	43.7 (39.6, 47.8)		25.9 (22.8, 29.0)	1.0
2010										
Middle-school graduate or less	1145.8 (1128.5, 1163.1)	3.2 (3.1, 3.3)	337.9 (329.1, 346.8)	2.1 (2.0, 2.2)	71.7 (67.5, 75.8)	3.3 (2.9, 3.7)	79.8 (75.3, 84.3)	2.3 (2.0, 2.5)	114.8 (109.0, 120.6)	3.3 (3.0, 3.6)
High-school graduate	556.9 (547.6, 566.2)	1.6 (1.6, 1.7)	209.4 (203.5, 215.2)	1.3 (1.3, 1.4)	34.6 (32.2, 36.9)	1.6 (1.4, 1.8)	45.2 (42.6, 47.9)	1.3 (1.2, 1.5)	52.8 (50.1, 55.6)	1.6 (1.4, 1.7)
College graduate or higher	370.9 (360.9, 381.0)	1.0	162.1 (155.3, 168.9)	1.0	21.3 (18.9, 23.8)	1.0	35.6 (32.4, 38.7)	1.0	35.1 (32.1, 38.0)	1.0

**Table 3:** **Age-adjusted mortality and relative risks of all-cause and four causes of death according to educational level in women**

	**All-cause**		**Malignant neoplasms**		**Cerebrovascular**		**Heart disease**		**Suicide**	
	**Mortality (95% CI)**	**RR (95% CI)**	**Mortality (95% CI)**	**RR (95% CI)**	**Mortality (95% CI)**	**RR (95% CI)**	**Mortality (95% CI)**	**RR (95% CI)**	**Mortality (95% CI)**	**RR (95% CI)**
30 ~ 44 years old										
1995										
Middle-school graduate or less	177.5 (171.3, 183.7)	3.5 (3.1, 3.8)	47.3 (44.2, 50.4)	2.3 (2.0, 2.7)	11.7 (10.2, 13.2)	5.8 (3.5, 9.7)	11.7 (10.1, 13.3)	6.2 (3.8, 10.2)	12.6 (10.9, 14.4)	2.7 (1.9, 3.7)
High-school graduate	73.1 (69.6, 76.5)	1.4 (1.3, 1.6)	27.6 (25.5, 29.8)	1.4 (1.2, 1.6)	5.2 (4.2, 6.1)	2.6 (1.6, 4.4)	2.8 (2.1, 3.5)	1.5 (0.9, 2.5)	4.7 (3.8, 5.5)	1.0 (0.7, 1.4)
College graduate or higher	54.8 (49.2, 60.4)	1.0	21.1 (17.6, 24.6)	1.0	2.3 (1.0, 3.7)	1.0	1.9 (0.7, 3.0)	1.0	4.7 (3.1, 6.3)	1.0
2000										
Middle-school graduate or less	220.1 (209.3, 231.0)	4.2 (3.8, 4.5)	54.3 (49.3, 59.4)	2.2 (1.9, 2.6)	13.6 (11.1, 16.1)	6.7 (4.4, 10.3)	12.6 (10.0, 15.3)	6.3 (4.1, 9.9)	27.2 (23.1, 31.2)	4.9 (3.8, 6.4)
High-school graduate	71.7 (68.7, 74.6)	1.5 (1.4, 1.7)	28.6 (26.7, 30.4)	1.3 (1.1, 1.5)	4.7 (3.9, 5.4)	2.5 (1.6, 3.8)	2.3 (1.8, 2.9)	1.4 (0.9, 2.1)	7.0 (6.1, 7.9)	1.5 (1.1, 1.9)
College graduate or higher	47.8 (43.9, 51.6)	1.0	22.5 (19.8, 25.2)	1.0	1.9 (1.1, 2.7)	1.0	1.4 (0.7, 2.1)	1.0	5.2 (3.9, 6.4)	1.0
2005										
Middle-school graduate or less	332.1 (308.8, 355.4)	6.1 (5.6, 6.7)	80.1 (69.5, 90.7)	3.3 (2.8, 3.7)	16.9 (12.6, 21.1)	8.5 (5.8, 12.5)	15.5 (10.8, 20.1)	23.5 (13.1, 42.4)	64.6 (53.8, 75.5)	5.6 (4.6, 6.7)
High-school graduate	72.1 (69.2, 75.0)	1.6 (1.5, 1.8)	26.7 (24.9, 28.5)	1.3 (1.1, 1.4)	3.7 (3.1, 4.4)	2.1 (1.5, 3.0)	1.9 (1.4, 2.4)	3.2 (1.8, 5.7)	15.9 (14.5, 17.3)	1.7 (1.4, 1.9)
College graduate or higher	45.4 (42.6, 48.3)	1.0	21.5 (19.5, 23.6)	1.0	1.4 (0.8, 2.0)	1.0	0.5 (0.1, 0.8)	1.0	9.4 (8.1, 10.6)	1.0
2010										
Middle-school graduate or less	377.5 (344.5, 410.6)	6.8 (6.2, 7.5)	57.6 (46.5, 68.7)	2.8 (2.3, 3.3)	22.0 (14.7, 29.3)	12.6 (8.4, 19.2)	8.4 (4.5, 12.4)	10.0 (5.7, 17.6)	121.3 (101.2, 141.5)	6.7 (5.7, 8.0)
High-school graduate	84.3 (80.8, 87.8)	1.8 (1.7, 2.0)	25.3 (23.4, 27.1)	1.3 (1.1, 1.4)	3.7 (3.0, 4.5)	2.6 (1.8, 3.7)	1.9 (1.4, 2.4)	2.1 (1.3, 3.4)	28.1 (26.0, 30.2)	1.9 (1.7, 2.1)
College graduate or higher	46.8 (44.3, 49.4)	1.0	20.1 (18.4, 21.8)	1.0	1.4 (0.9, 1.9)	1.0	0.5 (0.1, 0.8)	1.0	15.0 (13.6, 16.4)	1.0
45 ~ 59 years old										
1995										
Middle-school graduate or less	485.3 (477.8, 492.7)	1.8 (1.6, 1.9)	160.1 (155.8, 164.4)	1.3 (1.1, 1.5)	93.5 (90.3, 96.7)	2.6 (1.9, 3.4)	35.1 (33.1, 37.1)	2.1 (1.4, 3.2)	8.1 (7.1, 9.2)	2.6 (1.1, 5.8)
High-school graduate	321.7 (304.7, 338.6)	1.1 (1.0, 1.3)	136.7 (125.8, 147.6)	1.1 (0.9, 1.2)	53.9 (46.7, 61.0)	1.5 (1.1, 2.0)	19.8 (15.5, 24.1)	1.2 (0.7, 1.8)	6.1 (3.8, 8.4)	1.8 (0.8, 4.3)
College graduate or higher	303.4 (269.7, 337.0)	1.0	134.7 (112.7, 156.6)	1.0	37.6 (25.7, 49.5)	1.0	18.3 (9.6, 27.0)	1.0	4.1 (0.3, 7.9)	1.0
2000										
Middle-school graduate or less	399.9 (393.2, 406.6)	1.8 (1.7, 2.0)	140.8 (136.8, 144.7)	1.1 (1.0, 1.2)	67.6 (64.9, 70.3)	4.1 (3.0, 5.7)	29.5 (27.7, 31.3)	2.5 (1.7, 3.6)	10.7 (9.5, 11.8)	2.4 (1.4, 4.3)
High-school graduate	272.4 (260.0, 284.7)	1.2 (1.1, 1.3)	122.0 (113.7, 130.2)	0.9 (0.8, 1.1)	34.6 (30.0, 39.1)	2.1 (1.5, 3.0)	22.4 (18.6, 26.1)	1.7 (1.1, 2.5)	6.1 (4.4, 7.8)	1.4 (0.8, 2.6)
College graduate or higher	229.2 (207.7, 250.7)	1.0	128.6 (112.8, 144.3)	1.0	19.8 (12.9, 26.7)	1.0	13.7 (8.1, 19.4)	1.0	3.6 (1.1, 6.0)	1.0
2005										
Middle-school graduate or less	339.9 (333.5, 346.4)	2.0 (1.9, 2.2)	140.2 (136.1, 144.4)	1.4 (1.3, 1.6)	41.7 (39.4, 43.9)	3.0 (2.4, 3.9)	19.8 (18.3, 21.4)	2.3 (1.6, 3.1)	20.8 (19.2, 22.5)	2.0 (1.6, 2.7)
High-school graduate	221.0 (212.5, 229.6)	1.3 (1.2, 1.4)	110.8 (104.8, 116.8)	1.1 (1.0, 1.2)	24.4 (21.5, 27.3)	1.7 (1.3, 2.3)	11.7 (9.7, 13.7)	1.3 (0.9, 1.8)	12.2 (10.3, 14.1)	1.1 (0.8, 1.5)
College graduate or higher	176.3 (162.9, 189.7)	1.0	103.7 (93.4, 113.9)		14.2 (10.3, 18.1)	1.0	10.2 (6.8, 13.6)	1.0	10.2 (7.2,13.1)	1.0
2010										
Middle-school graduate or less	299.8 (292.9, 306.7)	2.0 (1.9, 2.1)	126.5 (122.1, 130.9)	1.4 (1.3, 1.5)	26.9 (24.9, 28.9)	3.2 (2.5, 4.1)	6.8 (15.2, 18.4)	3.2 (2.3, 4.4)	26.4 (24.2, 28.6)	1.8 (1.5, 2.2)
High-school graduate	180.9 (175.1, 186.7)	1.2 (1.1, 1.3)	92.0 (87.8, 96.1)	1.0 (0.9, 1.1)	16.3 (14.5, 18.0)	1.9 (1.5, 2.5)	8.1 (6.8, 9.4)	1.6 (1.1, 2.2)	16.3 (14.6, 17.9)	1.1 (0.9, 1.3)
College graduate or higher	153.5 (144.1, 162.8)	1.0	94.0 (86.6, 101.4)	1.0	9.1 (6.8, 11.5)	1.0	5.6 (3.7, 7.5)	1.0	14.7 (12.1, 17.4)	1.0

An increase in inequality was observed in women aged 30–44 years, especially among those with the lowest education level (Table 3). In 1995, the RRs for total mortality in women were 3.5 and 1.4 among those with the lowest educational level and high school graduates, respectively. However, the RRs for total mortality increased in 2010 to 6.8 for those with the lowest educational level and to 1.8 for high school graduates. These trends were most prominent in terms of suicide. The RR of suicide for middle school graduates or less increased from 2.7 in 1995 to 8.09 in 2010. Similarly, cerebrovascular and heart disease also showed substantial inequalities. In 2010, the RRs of those with a middle school education or less were 12.6 for cerebrovascular disease and 10.0 for heart disease.

Table 3 shows a striking increase in total mortality rates among younger women in the lower education group, a trend not seen in either men or older women. The all-cause mortality rate of younger women in the lowest education level increased from 177.5 per 100,000 in 1995 to 377.5 per 100,000 in 2010. The mortality rates for suicide increased across all educational levels over time, similar to that seen in men and older women. However, the mortality rates for suicide in younger women with the lowest education level increased dramatically from 12.6 per 100,000 in 1995 to 121.3 per 100,000 in 2010, a nearly tenfold increase in the span of 15 years. In 2010, suicide was the most common cause of death in younger women with a lower education level.

The magnitude of the RR for mortality was lower among older adults relative to their younger counterparts (Tables 2 and 3). With the exception of suicide, increased inequalities were also found for total mortality and three other leading causes of death in both older men and women. Among those who middle school graduate or less, the greatest increases in RRs were for cerebrovascular disease in men (1.9 vs. 3.3, respectively) and heart disease in women (2.1 vs. 3.2, respectively). Among women, the RR for suicide decreased in both lower education level groups, although mortality rates for suicide increased in all education level groups over time.

**Figure 1:**
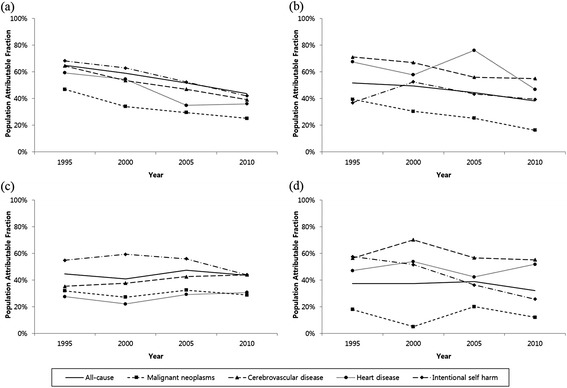
**The population attributable fraction of low education for all-cause mortality and four causes of death, 1995 ~ 2010. (a)** men aged 30–44, **(b)** women aged 30–44, **(c)** men aged 45–59, and **(d)** women aged 45–59.

Figure 1 and Additional file 1 show changes in the PAF of low education for mortality during the study period by gender and age. The PAF of low education level for total mortality has decreased since 1995 in both genders and age groups, although this trend was more prominent among those aged 30–44 years. The PAF of low education decreased by more than 20% points for total mortality as well as four leading causes of death between 1995 and 2010 for men aged 30–44 years. As of 2010, the PAFs of low education for total mortality were 43.7% for men and 38.3% for women aged 30–44 years. Among the four leading causes of death, the PAFs of low education were highest for suicide and cerebrovascular disease in men and for cerebrovascular and heart disease in women.

While the PAFs of low education for most leading causes of death decreased during study period, increasing trends in PAFs were also observed, including suicide in younger women, cerebrovascular disease in older men, and heart disease in both older men and older women. Among older men, the PAF of low education for cerebrovascular disease and heart disease increased by 8.7 and 3.1% points, respectively. The PAF of low education for heart disease increased by 4.8% points among older women, while the PAF for suicide increased by 2.4% points among younger women.

## Discussion

In 2010, the PAFs of low education for total mortality were 43.7% and 38.3% in men and women aged 30–44 years, respectively, compared with 43.5% and 32.0% in men and women aged 45–59 years, respectively. This means that if all adults aged 30 years old and over enjoyed the advantages of those with a college education or higher, approximately 32-44% of all deaths could be prevented.

In the United States [25], 11.5% of total mortality was attributed to a low education level in 2000; this figure is 20-30% points lower than the comparable figure in our study. Much of this difference can be ascribed to differences in the definition of low education level. The United States study divided education level into two groups (< high school, ≥ high school diploma or equivalent), and the group with at least a high school education was used as the theoretical minimum risk group. In contrast, we divided education level into three groups (middle school graduate or less, high school graduate, college graduate or higher), the latter of which was used as the counterfactual group. If we had used the same definition of low education level as that used in the U.S. study (< high school, ≥ high school diploma or equivalent), one-fifth of our study subjects would have fallen into the less-educated group, which may have reduced the PAF.

The PAF of low education for total mortality decreased in our study, even though the RRs for total mortality increased over the same period. The reduction in the PAF of low education was mainly due to the improvement in educational attainment in both genders and age groups. Educational opportunities in Korea have steadily increased across levels of study, including elementary, secondary, and tertiary education [10]. In 1945, when Korea was liberated from Japanese colonial rule, only 65% of primary school-aged children were enrolled in school, and the enrollment rate for secondary schools was less than 20%. Thanks to the strong government commitment to the “six years of compulsory primary plan,” the enrollment rate for elementary school increased to 96.4% in 1959, and the goal of universal primary education was fulfilled around the early 1960s [26]. In the 1970s, the implementation of the middle school screening system without examination and the introduction of a high school standardization system provided an opportunity for the spread of secondary education. By 1970, 66% of primary school graduates entered middle school, and 70% of middle school students continued on to high school. In the 1980s and 1990s, college enrollment increased substantially as a result of changes in government policies related to the relaxation of entrance quotas. By 1999, almost all students graduating from elementary and middle schools continued on to the next level of schooling, with 85% of graduates from academic high schools continuing on to higher education. This substantial improvement in educational attainment in Korea can be attributed to state-led educational reforms and a parental emphasis on their children’s education derived from the importance placed by Confucianism on scholarship and education [26-29].

Individuals born between 1967 and 1981 have received the greatest benefits as a result of changes in Korea’s educational policies. Further reflecting changes in social attitudes regarding gender equality, the increase in the proportion of college graduates was higher among women than men aged 30–44 years from 1995 to 2010. However, the most prominent decrease in the PAFs of low education for total mortality was observed among men, not women, during this period. The relatively low reduction in the PAF of low education among women despite greater improvements of educational attainment relative to their male counterparts was due to the ascending trend in RRs among those with low education. Total mortality inequality among Korean women between the ages of 25 and 44 also increased, a difference primarily attributable to changes in suicide rates within this group [18]. The suicide rate for women aged 30–44 years showed an increase in both the RR and PAF for low education groups, consistent with that seen in previous studies. During the financial crisis, the RR for suicide among those with the lowest degree of educational attainment increased in women aged 30–44 years [30], with the strongest effects on mortality seen in those aged 34–44 years [15]. Taken together, these data show that as educational attainment improved, women with the lowest degree of education attainment were at the greatest disadvantage in many respects. Social policies aimed at further helping these disadvantaged individuals are therefore urgently needed.

Note that the suicide rate among individuals in the lowest educational group increased about tenfold between 1995 and 2010 in women aged 30–44 years. Korea’s suicide rate was the highest among OECD countries for 10 consecutive years beginning in 2002 [31], characterized by a relatively low gender ratio (men vs. women), especially among younger adults [32]. Suicide rates among women aged 25–44 years are also the highest among five Asian countries—Korea, Hong Kong, Japan, Singapore, and Taiwan—and have remained that way since 1997 [33]. Factors contributing to these relatively high suicide rates in women include the feminization of poverty and labor market marginalization, especially in women with a low education level [34].

Among older age groups, the PAF of low education for cerebrovascular and heart diseases in men, and for heart disease in women, increased by 3-9% points between 1995 and 2010. This increase was due, in part, to the widening of inequalities in terms of disease-specific mortality. Many studies have reported inequalities in mortality related to cardiovascular disease (CVD), while many others have sought to identify the root causes underlying these inequalities [35-37]. The magnitude and direction of educational inequalities in CVD mortality varied among the United States and 11 European countries, as each country was characterized by a different distribution of cardiovascular risk factors such as cigarette smoking, alcohol consumption, obesity, and lack of fresh vegetables [35]. Numerous studies have reported socioeconomic inequalities in health behaviors related to CVD in Korea [38-42]. In particular, cigarette smoking was the leading factor driving inequality in CVD mortality among men aged 30–64 years [43]. The smoking rate of Korean men aged >15 years of age was the second highest among OECD countries at 48.4% in 2010 [44]. These rates are even more pronounced when stratified based on education, with 64.7% of men aged 25–64 years having a middle school education or less describing themselves as smokers compared to 49.1% of individuals with a college degree or higher. Moreover, the RIIs of current cigarette smoking increased from 1995 to 2006 among both men and women [45], which may have contributed to the increase in the RR of low education for mortality related to CVD. One of the most important factors in the treatment of CVD is time from symptom onset to treatment, and a study conducted in South Korea found inequality in the access to emergency medical services according to education level [46], which may also contribute to the widening of inequalities in terms of CVD mortality.

The PAF is composed of three elements: the RRs for each cause of death as a function of exposure level, the current levels of exposure, and the counterfactual distribution of exposure. According to the equation for the PAF, the accuracy and relevance of the estimated PAF depend on these three elements. Thus, both the limitations and strengths of our study can be discussed in this context.

First, because PAF research usually estimates RRs based on case–control or prospective cohort studies, information about the RRs for every risk factor-disease pair is difficult to obtain. Consequently, RRs are assumed to be universal, and the RR from one population has been applied to many other populations, albeit cautiously [43]. However, the RR may differ across populations and time periods, which can be characterized by varying exposure levels. In this study, we directly calculated the RRs of disease-specific mortality rates by age, gender, and time period based on nationally representative data rather than by applying the RRs of other populations. Furthermore, we considered differences according to the level of exposure. We divided the lower education level into two groups, middle school graduates or less and high school graduates, because those with a middle school education or less are expected to have a higher risk for mortality than high school graduates. These methods allowed us to estimate the PAF of low education more precisely. However, a numerator-denominator bias may affect education-specific mortality rates based on unlinked data for the numerator and the denominator, possibly yielding biased educational mortality differentials. Second, in terms of current levels of exposure, we chose periods of analysis based on census years, and the distribution of the population in terms of educational level was estimated directly for a given census year. Since a census is an official survey of the population of a country, it is suitable for estimating the current levels of educational attainment of a population. This approach constitutes a strength of this study. Third, with regard to the counterfactual distribution of exposure, education, which was used as the indicator of SEP, is an ordinal variable. Thus, the theoretically minimum risk distribution would be found among those who had achieved the highest level of education, a doctorate. However, we chose to use the group with at least a college education as reflective of the theoretically minimum risk distribution because of limitations in the available data for levels higher than college graduation. As a result, we might have underestimated the PAF of low education.

## Conclusions

A consistent and sharp increase in educational attainment contributed to the decrease in PAFs of low education for mortality despite the lack of improvement in mortality inequalities. These results clearly demonstrate the importance of socioeconomic factors, such as education, as a means of improving health inequalities, indicating the need for a more comprehensive approach to public health policy. Expanding access to basic education in developing countries may therefore provide additional health benefits, while in developed countries, efforts must be taken to narrow the gap in terms of access to higher education, as a means to combat chronic public health issues.

Although the proportion of the population with low education and the magnitude of the PAFs of low education for mortality have decreased, the RR of mortality for those with the lower educational level has increased. Policies to assist those in the most disadvantaged socioeconomic position are urgently needed, as this group is shrinking and growing weaker.

## Additional file

Additional file 1:
**The population attributable fraction of low education for mortality in South Korea (1995 ~ 2010).**

## References

[1] Mackenbach JP, Bos V, Andersen O, Cardano M, Costa G, Harding S (2003). Widening socioeconomic inequalities in mortality in six Western European countries. Int J Epidemiol.

[2] Mackenbach JP, Stirbu I, Roskam A-JR, Schaap MM, Menvielle G, Leinsalu M (2008). Socioeconomic inequalities in health in 22 European Countries. N Engl J Med.

[3] Huisman M, Kunst AE, Bopp M, Borgan J-K, Borrell C, Costa G (2005). Educational inequalities in cause-specific mortality in middle-aged and older men and women in eight western European populations. Lancet.

[4] Spiegelman D, Hertzmark E, Wand HC (2007). Point and interval estimates of partial population attributable risks in cohort studies: examples and software. Cancer Causes Control.

[5] Galobardes B, Shaw M, Lawlor DA, Smith GD, Lynch J, Oakes JM, Kaufman JS (2006). Indicators of socioeconomic position. Methods in social epidemiology.

[6] Ljung R, Peterson S, Hallqvist J, Heimerson L, Diderichsen F (2005). Socioeconomic differences in the burden of disease in Sweden. Bull World Health Organ.

[7] Kulhánová I, Hoffmann R, Judge K, Looman CW, Eikemo TA, Bopp M (2014). Assessing the potential impact of increased participation in higher education on mortality: evidence from 21 European populations. Soc Sci Med.

[8] Galobardes B, Shaw M, Lawlor DA, Lynch JW, Davey SG (2006). Indicators of socioeconomic position (part 1). J Epidemiol Community Health.

[9] Telfair J, Shelton TL (2012). Educational attainment as a social determinant of health. N C Med J.

[10] Birger F, Jee Peng T, Lee J-J, Adams D, Kim S-Y, Kim S-K, Jung J-Y, Cho N-S, World Bank (2009). An African exploration of the East Asian education experience. Korea Education '60, Achievements and Challenges.

[11] Yang S, Khang YH, Harper S, Davey Smith G, Leon DA, Lynch J (2010). Understanding the rapid increase in life expectancy in South Korea. Am J Public Health.

[12] Son M, Oh J, Choi YJ, Kong JO, Choi J, Jin E (2006). The effects of the parents’ social class on infant and child death among 1995–2004 birth cohort in Korea. J Prev Med Public Health.

[13] Cho HJ, Khang YH, Yang S, Harper S, Lynch JW (2007). Socioeconomic differentials in cause-specific mortality among South Korean adolescents. Int J Epidemiol.

[14] Khang YH, Kim HR (2006). Socioeconomic mortality inequality in Korea: mortality follow-up of the 1998 National Health and Nutrition Examination Survey (NHANES) data. J Prev Med Public Health.

[15] Khang YH, Lynch JW, Kaplan GA (2004). Health inequalities in Korea: age- and sex-specific educational differences in the 10 leading causes of death. Int J Epidemiol.

[16] Jeong BG, Jung KY, Kim JY, Moon OR, Lee YH, Hong YS (2006). The relationship between regional material deprivation and the standardized mortality ratio of the community residents aged 15–64 in Korea. J Prev Med Public Health.

[17] KIHASA (2013). Developing Health Inequalities Indicators and Monitoring the Status of Health Inequalities in Korea.

[18] Jung-Choi K, Khang YH, Cho HJ (2011). Changes in contribution of causes of death to socioeconomic mortality inequalities in Korean adults. J Prev Med Public Health.

[19] KOSIS. Population Census. 1995, 2000, 2005, 2010. http://kosis.kr. Accessed 25 Jun 2014.

[20] Kwon TH, Kim TH (1990). Life table in Korea, 1970–1985.

[21] Kim HR, Khang YH (2005). Reliability of education and occupational class: a comparison of health survey and death certificate data. J Prev Med Public Health.

[22] OECD. Classifying educational Programmes, Manual for ISCED-97 implementation in OECD Countries 1999 Edition. 1999. http://www.oecd.org/edu/1841854.pdf. Accessed 25 Jun 2014.

[23] Vander Hoorn S, Ezzati M, Rodgers A, Lopez AD, Murray CJL, Ezzati M, Lopez AD, Rodgers A, Murray CJL (2004). Estimating attributable burden of disease from exposure and hazard data. Comparative quantification of health risks: global and regional burden of disease attributable to selected major risk factors.

[24] WHO (2013). Handbook on health inequality monitoring: with a special focus on low- and middle-income countries.

[25] Galea S, Tracy M, Hoggatt KJ, DiMaggio C, Karpati A (2011). Estimated death attributable to social factors in the United States. Am J Public Health.

[26] Kim S, Lee J-H (2010). Private tutoring and demand for education in South Korea. Economic Development and Cultural Change.

[27] Lee S, Brinton MC (1996). Elite education and social capital: the case of South Korea. Sociol Educ.

[28] Sorensen CW. Success and education in South Korea. Comparative Educ Rev. 1994;38(1):10–35.

[29] Seth MJ (2002). Education fever: society, politics, and the pursuit of schooling in South Korea.

[30] Lee WY, Khang YH, Noh M, Ryu JI, Son M, Hong YP (2009). Trends in educational differentials in suicide mortality between 1993–2006 in Korea. Yonsei Med J.

[31] OECD iLibrary. OECD health data: health status. http://www.oecd-ilibrary.org. Accessed 26 Jan 2015.

[32] Hee Ahn M, Park S, Ha K, Choi SH, Hong JP (2012). Gender ratio comparisons of the suicide rates and methods in Korea, Japan, Australia, and the United States. J Affect Disord.

[33] Hong J. Socio-economic inequalities in mental health and their determinants in South Korea. PhD thesis, The London School of Economics and Political Science. 2012.

[34] Kim MH, Jung-Choi K, Jun HJ, Kawachi I (2010). Socioeconomic inequalities in suicidal ideation, parasuicides, and completed suicides in South Korea. Soc Sci Med.

[35] Mackenbach JP, Cavelaars AE, Kunst AE, Groenhof F (2000). Socioeconomic inequalities in cardiovascular disease mortality; an international study. Eur Heart J.

[36] Kamphuis CB, Turrell G, Giskes K, Macenbach JP, Lenthe FJ (2012). Socioeconomic inequalities in cardiovascular mortality and the role of childhood socioeconomic conditions and adulthood risk factors: a prospective cohort study with 17-years of follow up. BMC Public Health.

[37] Strand BH, Tverdal A (2004). Can cardiovascular risk factors and lifestyle explain the educational inequalities in mortality from ischaemic heart disease and from other heart diseases? 26 year follow up of 50,000 Norwegian men and women. J Epidemiol Community Health.

[38] Khang YH, Cho HJ (2006). Socioeconomic inequality in cigarette smoking: trends by gender, age, and socioeconomic position in South Korea, 1989–2003. Prev Med.

[39] Cho HJ, Song YM, Smith GD, Ebrahim S (2004). Trends in socio-economic differentials in cigarette smoking behaviour between 1990 and 1998: a large prospective study in Korean men. Public Health.

[40] Yoon TH, Moon OR, Lee SY, Jeong BG, Lee SJ, Kim NS (2000). Differences in health behaviors among the social strata in Korea. J Prev Med Public Health.

[41] Yang YJ, Yoon YS, Oh SW, Lee ES (2005). The amount of physical activity of Korean adults measured from the 2001 KNHANES. Korean J Fam Med.

[42] Yoon YS, Oh SW, Park HS (2006). Socioeconomic status in relation to obesity and abdominal obesity in Korean adults: a focus on sex differences. Obesity.

[43] Khang YH, Lynch JW, Jung-Choi K, Cho HJ (2008). Explaining age-specific inequalities in mortality from all causes, cardiovascular disease and ischaemic heart disease among South Korean male public servants: relative and absolute perspectives. Heart.

[44] OECD iLibrary. Non-Medical Determinants of Health: tobacco consumption. http://www.oecd-ilibrary.org. Accessed 26 Jan 2015.

[45] Khang YH, Yun SC, Cho HJ, Jung-Choi K (2009). The impact of governmental antismoking policy on socioeconomic disparities in cigarette smoking in South Korea. Nicotine Tob Res.

[46] Park J. Accessibility of Emergency Medical Services (EMS) to acute myocardial infarction by educational level. Master’s thesis, Korea University Graduate School of public health. 2011.

