# Supplementary material for: The population attributable fraction of low education for mortality in South Korea with improvement in educational attainment and no improvement in mortality inequalities
Source: BMC Public Health. 2015 Mar 31;15:313. doi: 10.1186/s12889-015-1665-x (PMC4425894; doi:10.1186/s12889-015-1665-x)
Supplement: Additional file 1: — The population attributable fraction of low education for mortality in South Korea (1995 ~ 2010). [file 12889_2015_1665_MOESM1_ESM.pdf]

Additional file 1. The population attributable fraction of low education for mortality in South Korea (1995~2010)

(%)

	1995		2000		2005		2010	
	PAF	(95% CI)	PAF	(95% CI)	PAF	(95% CI)	PAF	(95% CI)
Men								
30~44 years								
All-cause	65.0	(59.0, 70.9)	58.9	(52.8, 65.1)	51.8	(44.8, 58.7)	43.7	(37.6, 49.9)
Malignant neoplasms	46.9	(37.1, 56.8)	34.0	(26.2, 41.8)	29.6	(21.9, 37.3)	25.2	(18.4, 31.9)
Cerebrovascular	64.6	(38.7, 90.4)	53.5	(31.3, 75.7)	47.0	(24.9, 69.2)	39.1	(18.2, 60.0)
Heart disease	59.3	(16.2, 100[b])	54.6	(34.1, 75.0)	34.9	(20.4, 49.4)	36.0	(16.8, 55.3)
Suicide	68.3	(41.8, 94.8)	62.9	(40.8,85.0)	52.4	(35.2, 69.7)	42.0	(31.3, 52.8)
45~59 years								
All-cause	44.5	(38.4, 43.0)	40.7	(38.4, 43.0)	47.4	(45.1, 49.6)	43.5	(41.5, 45.6)
Malignant neoplasms	31.9	(23.2, 31.1)	27.1	(23.2, 31.1)	32.3	(28.9, 35.7)	28.8	(25.8, 31.7)
Cerebrovascular	35.2	(30.6, 44.7)	37.6	(30.6, 44.7)	42.5	(35.2, 49.8)	43.9	(35.6, 52.3)
Heart disease	27.5	(13.8, 30.2)	22.0	(13.8, 30.2)	29.2	(22.1, 36.2)	30.6	(24.3, 36.9)
Suicide	54.7	(44.7, 73.8)	59.2	(44.7, 73.8)	55.9	(46.2, 65.6)	43.9	(37.1, 50.7)
Women								
30~44 years								
All-cause	51.7	(43.7, 59.6)	49.4	(41.9, 57.0)	44.6	(35.7, 53.4)	38.3	(30.3, 46.3)
Malignant neoplasms	39.4	(26.7, 52.0)	30.5	(20.7, 40.2)	25.3	(16.5, 34.0)	16.3	(10.1, 22.5)
Cerebrovascular	71.2	(32.5, 100[b])	67.1	(27.2, 100[b])	56.1	(8.9, 100[b])	55.0	(0[a],100[b])
Heart disease	67.5	(23.2, 100[b])	57.7	(12.2, 100[b])	76.2	(0[a],100[b])	46.9	(0[a],100[b])
Suicide	37.0	(7.0, 67.0)	52.5	(26.4, 78.6)	43.5	(23.9, 63.0)	39.4	(24.1, 54.7)
45~59 years								
All-cause	37.4	(0[a],100[b])	37.3	(29.5, 45.1)	38.7	(32.8, 44.5)	32.0	(27.3, 36.8)
Malignant neoplasms	17.9	(0[a],100[b])	5.0	(0[a],19.0)	20.0	(10.8, 29.1)	12.0	(4.7, 19.3)
Cerebrovascular	56.4	(35.4, 77.4)	70.2	(50.4, 89.9)	56.6	(39.3, 73.8)	55.2	(38.8, 71.5)
Heart disease	46.9	(10.0, 83.9)	53.7	(26.8, 80.6)	42.3	(17.0, 67.5)	51.7	(29.4, 74.1)
Suicide	57.5	(2.5, 100[b])	51.5	(9.0, 94.1)	36.2	(12.8, 59.7)	25.6	(10.7, 40.5)

[a] A value less than 0 was replaced by 0

[b] A value greater than 100 was replaced by 100
